# Relationship between caffeine intake and infertility: a systematic review of controlled clinical studies

**DOI:** 10.1186/s12905-020-00973-z

**Published:** 2020-06-16

**Authors:** Fan-Long Bu, Xue Feng, Xiao-Ying Yang, Jun Ren, Hui-Juan Cao

**Affiliations:** 1 Centre for Evidence-based Chinese Medicine, Beijing University of Chinese Medicine, 11, the 3rd Ring Road East, Chaoyang District, Beijing, 100029 China; 2 China Association of Chinese Medicine, Beijing, 100029 China; 3 Ineye Hospital of Chengdu University of TCM, Chengdu, 610036 China; 4 Beijing Cainiaohd Technology CO.,LTD, Beijing, 100124 China

**Keywords:** Caffeine, Coffee, Infertility, Systematic review, Meta-analysis

## Abstract

**Background:**

For a long time, the relationship between caffeine consumption and infertility in the general population is unclear, this study is aimed to systematically review the evidence from any type of controlled clinical studies to explore whether caffeine intake is a risk factor for human infertility.

**Methods:**

Seven databases were searched from inception to May 2019. We included women/men without a history of infertility but were willing to have children in prospective studies and women/men who were diagnosed with infertility in retrospective studies. The observed exposure factor should be caffeine or caffeine containing beverage. Diagnosis of infertility or not for participants was the key outcome. The Newcastle-Ottawa scale (NOS) or Cochrane risk of bias tool were used to assess the methodological quality of included studies. Meta-analysis was conducted if there were acceptable clinical and statistical heterogeneity among studies. The GRADE method was used to assess the certainty of the evidence.

**Results:**

Four studies (one cohort study and three case-control studies) involving 12,912 participants were included. According NOS, the average score of case-control studies was 6, and the cohort study achieved 9. Meta-analysis and subgroup analysis were conducted. The results showed that low (OR 0.95, 95%CI 0.78–1.16), medium (OR 1.14, 95%CI 0.69–1.86) and high doses (OR 1.86, 95%CI 0.28–12.22) of caffeine intake may not increase the risk of infertility. The quality of the current evidence bodies were all low.

**Conclusion:**

Our study provides low quality evidence that regardless of low, medium and high doses of caffeine intake do not appear increase the risk of infertility. But the conclusion should be treated with caution.

## Background

As a reproductive system disease, infertility is defined as the failure to achieve a clinical pregnancy after at least 12 months of routine unprotected sexual intercourse, except for reasons such as breast feeding or postpartum menstruation [1]. Primary infertility is defined as no live birth for women who want to have a child and have been married for at least 5 years without using any contraceptive measures during this period [2]. Many biological factors and other causes may lead to infertility, including some interventional reasons due to treatment [3]. In vitro fertilization (IVF) appears to be the first choice for infertile couples without the recognition of effective treatments, which may result in approximately 30% of live births [4].

Caffeine is widely present in many beverages and foods, especially tea, coffee, cola, energy drinks and chocolate [5]. Effects of caffeine on health is like a double-edged sword. It may have a protective effect on cardiovascular diseases (such as coronary heart disease, arrhythmia, and heart failure), diabetes, liver disease [6], and even Parkinson’s disease [7]. For the negative effects of the reproductive system, one review [8] showed that per 100 mg/d caffeine intake may increase the risk of miscarriage (RR 1.14,95%CI 1.10–1.19), stillbirth (RR 1.19,95%CI 1.05–1.35), premature delivery (RR 1.02,95%CI 0.98–1.06), low birth weight (RR 1.07,95%CI 1.01–1.12) and small for gestational age (SGA) infants (RR 1.10,95%CI 1.06–1.14).

For a long time, the relationship between caffeine consumption and infertility in the general population is unclear, and different studies often draw opposite conclusions [9–16]. We searched only one related systematic review, it [9] showed that caffeine intake may have a negative impact on male reproductive function, but the relationship between caffeine intake and semen parameters or male fertility has not been found in published literatures. In summary, determining the relationship between caffeine intake and infertility is crucial. Therefore, we conducted this study to systematically review evidence from any type of controlled clinical study to explore whether caffeine intake is a risk factor for human infertility.

## Methods

### Protocol and registration

The protocol was registered at PROSPERO international register of systematic reviews (No.CRD42015015714) on 25 December 2014 (Available from http://www.crd.york.ac.uk/PROSPERO/display_record.php?ID=CRD42015015714). After registration, we published the protocol in 2016 in Systematic Reviews [17]. During the submission and peer review, we made some revisions of the previous version of the protocol regarding the comments of the reviewers. Since the purpose of this review is to investigate the relationship between caffeine intake and infertility, we expanded the target population and type of studies as amendments. All the revisions had been pre-defined before performing the review.

### Eligibility criteria

Controlled clinical studies (CCT) were included, involving randomized controlled trials (RCTs), quasi-RCTs or non-randomized clinical studies (both prospective and retrospective), cohort studies, and case-control studies. There was no limitation on publication types or language.

We included women/men without a history of infertility but were willing to have children in prospective studies and women/men who were diagnosed with infertility in retrospective studies. All participants who met the inclusion criteria were of reproductive age and not menopause.

Coffee, tea, cola or other caffeinated beverages are often sources of caffeine in the daily diet. Only studies that observed caffeine or caffeinated beverages as exposure factors were included in this review, as caffeine contributed by caffeinated foods was small and caffeine doses were difficult to count.

Diagnosis of infertility or not for participants was the key outcome of this review. Generally accepted diagnostic criteria was mentioned in the original studies; according to which, those who were diagnosed as infertile had suffered from at least 12 months of unsuccessful conception.

### Search strategy

PubMed, the Cochrane CENTRAL Database, EMBASE, China National Knowledge Infrastructure (CNKI), Wanfang, VIP Database, and Chinese Biomedical Database (CBM) were searched from inception to May 2019. Unpublished literatures (such as conference report, dissertation, etc.) were achieved through CNKI, Wanfang database and CADTH Grey matters checklist (https://www.cadth.ca/resources/finding-evidence/grey-matters). Ongoing studies were also searched through the meta Register of Controlled Trials (http://www.controlled-trials.com), the US National Institutes of Health Ongoing Trials Register (www.clinicaltrials.gov), and the Australian New Zealand Clinical Trials Registry (www.anzctr.org.au). In order to avoid missing other relevant reports, all references of included studies were manually searched.

“Infertility” or “sterility” combined with “coffee”, “caffeinated” or “caffeine” were used for literature searching. Various morphology of caffeine, such as “coffein”,"calcium caffeine”, “caffeine calcium complex”, “anhydrous caffeine”, “cafeine”, “animine” and “caffein” were also used during the literature searching. The details of the search strategy are shown in Additional file 1.

### Study selection and data collection

NoteExpress software (version 3.2.0.7103) was used for the management of records downloaded from the databases and the selection of studies. Two authors (JR and XF) independently screening the literatures by reading the title and abstract. If a judgment could not be made, the full text was download and read. The third author (HJC) arbitrated if they could not reach a consensus.

A pre-designed screening table was used to double check the eligibility of potential included trials. Two authors (XY and XF) independently extracted relevant data according to the predefined data extraction table. Disagreement was resolved by HJC.

### Risk of bias in individual studies

Methodological quality of analytic studies was assessed according to the Newcastle-Ottawa Scale (NOS) [18] by two authors (FLB and XF), independently. Stars were awarded in the “representative” selection samples, “comparability” between groups, “completeness” and “validity” records of caffeine intake or infertility. The methodological quality of RCTs, quasi-RCTs, or nonrandomized clinical studies were planned to assess according to the Cochrane risk of bias tool [19]. Seven elements were assessed: random sequence generation, allocation concealment, blinding of included participants, blinding of outcome assessors, incomplete outcome data, selective reporting, and other biases.

### Summary measures and synthesis of results

Review Manager (Revman 5.3, 2014) was used to analyze the data. If data permit, dose of caffeine would be quantified on a daily basis. If daily caffeine consumption was not reported in milligrams, the following assumptions were used to estimate: one serving of coffee contained about 100 mg of caffeine, and any other caffeinated beverage (such as tea or cola) contained on average 60 mg of caffeine [4].Categorize caffeine doses based on daily consumption (mean or median). For individual studies, relative risk (including odds ratio, risk ratio, and hazard ratio) with their 95% confidence interval (CI) was measured for participants in the caffeine-intake and non-caffeine intake groups. Participants characteristics, exposure factors (such as dose of caffeine intake), and outcomes between studies constituted clinical heterogeneity. Statistical heterogeneity was tested by the *I*^2^ statistic and its 95% CI calculated [20]. Tau-squared and its 95% CI were further tested for differences between studies [21]. Meta-analysis was planned to do if there were acceptable clinical and statistical heterogeneity (*I*^2^ < 75%) among trials. Random effects models which were more conservative and provided better estimates with wider confidence intervals were planned to use when conducting meta-analysis [22]. Only data adjusted for the identical pre-specified confounders would be pooled in the meta-analysis. When the statistical heterogeneity was significant (*I*^2^ > 75%), subgroup analysis and sensitivity analysis mentioned below would be used to explore potential sources of heterogeneity. Even when reasonable, statistical heterogeneity might be ignored. When heterogeneity was significant and meta-analysis was not possible, forest plot without pooled steps were still presented. In this case, the synthesis of results was described qualitatively.

Subgroup analysis was intended to be conducted according to study design (retrospective or prospective), demographics (gender, age, history of infertility) and dose of caffeine intake. Dose-response was planned to be conducted if data permit. Sensitivity analysis was planned to be performed according to methodological quality (assessed by ROB or NOS) or study publication time (within 5 years or not).When more than 10 studies were included in one outcome, funnel plot were used to detect publication bias [23].

### Evidence assessment

The Grades of Recommendations Assessment, Development and Evaluation (GRADE) [24] was used to assess the quality of the evidence with meta-analysis. Considering the following aspects, such as methodological quality, outcome consistency of trials, directness and accuracy of evidence and possibility of publication bias, we judged whether to degrade the evidence of included randomized controlled trials, and assessed the level of the evidence as: high, moderate, low or very low. Level of the evidence from observational studies would be upgrade if the pooling results showed large effect, dose-response effect or would be impact by plausible confounding factors.

## Results

### Study selection

**Fig. 1:**
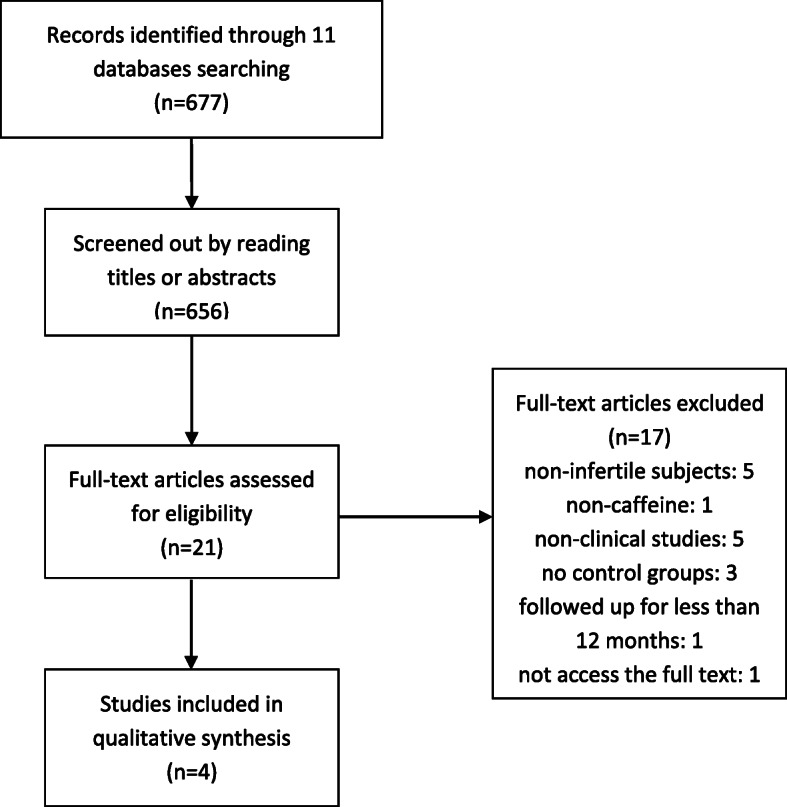
Flow chart for included studies

We searched the above 11 databases and obtained 677 literatures. After browsing the titles and abstracts, we downloaded 21 full texts. Finally, four studies [25–28] were included. Three were case-control studies and the remaining one was a cohort study, all of which were published in English. The flow chart is shown in Fig. 1.

### Characteristics of the included studies

A total of 12,912 participants were included in four studies [25–28]. Two of the four case-control studies [25, 27] observed male with abnormal sperm, such as dyspermia, azoospermia or oligospermia, and the other two involved female patients with primary infertility or minimal/mild endometriosis. The only cohort study [28] observed nulliparous female without a history of infertility. The exposures of two studies [25, 27] were coffee, and the other two were caffeine containing beverage (CCB), including caffeinated soft drinks, coffee, tea, cocoa or cola et al. For two of the studies [26, 28], exposure intensity were analyzed according to the daily caffeine intake (mg/d), and for the other two studies [25, 27] caffeine intake was calculated according to the corresponding conversion relationship of caffeine consumption according to each kind of CCB.

In three case-control studies [25–27], odds ratio (OR) were used to indicate the risk of infertility caused by coffee or caffeine intake. The cohort study [28] used hazard ratio (HR) to indicate the risk of caffeine intake. HR is a risk ratio that takes into account the time factors, that is, when the number of events is included in the dynamic cohort, it also takes into account the loss of follow-up or other exposure factors to cause the event to occur. In this review, OR and HR cannot be directly converted. The basic characteristics of the four studies included are shown in Table 1.

**Table 1:** Characteristics and results of included studies concerned caffeine intake with infertility

Study ID	Study Type	Participants			Exposure (Caffeine/coffee)	Comparison	Outcomes	
		Gender	Age	Sample size			Disease	Relative Risk(95%CI)
Buiatti 1984	Case-Control Study	Men	20-54y	239	≥100 mg/d	0 mg/d	Azoospermia/oligospermia	OR = 0.91 (0.46–1.82)
Grodstein 1993	Case-Control Study	Women	Unclear	4883	E_1_ 100-167mg/d; E_2_ 168-233mg/d; E_3_>233mg/d	<100 mg/d	Primary infertility	OR_1_ = 1.00(0.79–1.25); OR_2_ = 1.16(0.94–1.42); OR_3_ = 1.36(1.16–1.60) ^a,b^
Prazzini 1993	Case-Control Study	Men	Median 31-33y	216	E_1_ 200-300 mg/d; E_2_ ≥ 400 mg/d	0-100 mg/d	Infertile men with dyspermia	OR_1_ = 1.7(0.8–3.7); OR_2_ = 5.4 (2.4–12.6) ^c^
Liv2018	Cohort Study	Women	20-29y	7574	Coffee: E_1_ ≤ 200 ml/d E_2_ 200-400 ml/d E_3_ ≥ 500 ml/d	0 ml/d	Infertile women	HR_1_ = 0.86(0.70–1.06); HR_2_ = 0.88(0.73–1.06); HR_3_ = 0.89(0.72–1.10);
					Tea: E_1_ ≤ 200 ml/d E_2_ 200-400 ml/d E_3_ ≥ 500 ml/d	0 ml/d		HR_1_ = 1.10(0.84–1.44); HR_2_ = 1.10(0.84–1.46); HR_3_ = 1.15(0.87–1.53);
					Caffeine^d^: E_1_ ≤ 1-168 mg/d E_2_ 169-333mg/d E_3_ 334-579mg/d E_4_ ≥ 580 mg/d	0 mg/d		HR_1_ = 0.93(0.58–1.49); HR_2_ = 0.91(0.57–1.47); HR_3_ = 0.97(0.60–1.55); HR_4_ = 0.93(0.58–1.50)^e^

*Note: E* Exposure, *OR* Odds ratio, *HR* Hazard ratio, *CI* Confidence interval

^a^ This study classified the participants into 5 groups according to their primary disease and calculated the estimate effect of them respectively. Details of the information were reported in the main text of this review

^b^ OR were adjusted for center, age, lifetime number of sexual partners, current and former cigarette smoking, and alcohol intake

^c^ Age-Adjust OR

^d^ Total caffeine calculated from consumption of both coffee and tea

^e^ Educational level (≤9, 10–11 or ≥ 12 years of schooling), Smoking (yes or no), Marital status (married/cohabiting or single), Weekly alcohol intake and Year of birth-Adjust HR.

### Risk of bias of included studies

**Fig. 2:**
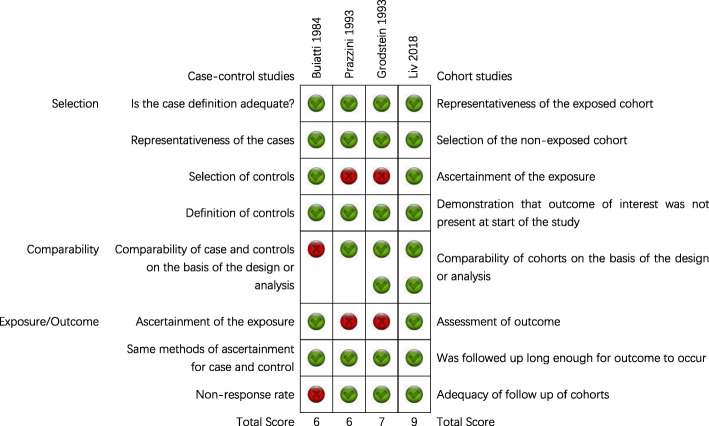
Quality evaluation of the included studies

The full score of NOS is 9 points. Only one case-control study [25] used blinded investigation, and the other three studies [26–28] used unblinded investigations or case records as a way to determine exposures or outcomes. Only one case-control study [25] used community controls, and the rest were hospital controls. One case-control study [25] did not describe non-response rate and did not adjust for confounding factors. Three case-control studies gained 6, 6 and 7 points respectively. The cohort study achieved 9 points. The methodological quality of the case-control studies included in this review is general, but the cohort study is good. The methodological quality of included studies is shown in Fig. 2.

### Estimate relationship between caffeine intake and infertility

#### For the effect of caffeine on male factors infertility

In Buiatti’s study [25], males with azoospermia or oligospermia (i.e., sperms/ml < 20, 000, 000) were used as the case group, and normal routine examinations and sperm counts > 20, 000, 000/ml were used as the control group in the same period. The study showed that drinking coffee seems to have no correlation with sperm abnormalities (RR 0.91, 95%CI 0.46–1.82). Although it was mentioned that the coffee intake was divided into different doses for further analysis, relevant data was not provided and the corresponding relative risk were not calculated based on them.

Prazzini’s study [27] had three groups, in which infertile men with dyspermia were used as the case group, and normospermic infertile men and fertile men with unknown semen quality as the control groups. Our study included only the case group and the fertile control group. With the number of cups of coffee per day, the risk of dyspermia increased. Compared to less than 100 mg/d caffeine intake those who had 200-300 mg/d caffeine intake may have an OR of 1.7 (95%CI 0.8–3.7), and those who had more than 400 mg/d caffeine intake may have even higher risk of infertility (OR 3.4, 95%CI 2.4–12.6).

#### For the effect of caffeine on female factors infertility

In Grodstein’s study [26], primary infertility was used as the case group and those with a history of delivery in the past 2 years as the control group. It reported the number of infertile patients with different doses of caffeine intake in different etiology. After adjusting for the relevant confounding factors, the risk of infertility caused by tubal disease (RR 1.5, 95%CI 1.1–2.0) increased significantly when caffeine consumption more than 7 g/m (233 mg/d), and the risk of cervical factors (OR 1.3, 95%CI 0.7–2.4, for 5.1–7 g/m and OR 1.4, 95%CI 0.9–2.3, for > 7 g/m) or endometriosis (OR 1.9, 95%CI 1.2–2.9, for 5.1–7 g/m and OR 1.6, 95%CI 1.1–2.4, for > 7 g/m) increased significantly when caffeine intake greater than 5 g/m (167 mg/d).We added the number of case together regardless the cause of infertility and re-analyzed the odds ratio. The results showed with 101-167 mg/d caffeine intake may not increase the risk of infertility compared to less 100 mg/d caffeine intake (OR 1.00, 95%CI 0.79–1.25), however, higher dose of caffeine intake may increase the risk (OR 1.16, 95%CI 0.94–1.42, for 168-233 mg/d; OR 1.36, 95%CI 1.16–1.60, for > 233 mg/d).

The only cohort study included [28] was a retrospective cohort study. It was divided into exposure and non-exposure group according to whether the participants drink coffee or tea. For different doses of total caffeine consumption (from coffee and tea), the risk of infertility for consumers was similar to that of never consumers. Only 100 mg/d of caffeine consumed did not affect the risk of primary infertility in the consumers (HR 1.00, 95% CI 0.98–1.02).

Details of the results from each single study was also shown in Table 1.

**Fig. 3:**
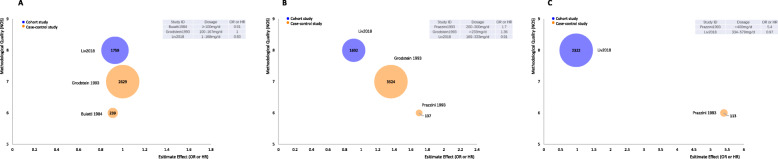
Evidence Mapping of caffeine intake for infertility. **a** Caffeine intake > 100 mg/d vs ≤100 mg/d. **b** Caffeine intake > 200 mg/d vs ≤100 mg/d. **c** Caffeine intake > 400 mg/d vs ≤100 mg/d

Considering the clinical heterogeneity among each study, we chose to present the single study’s results with bubble plot first. The X-axis represents the effect value (OR), the Y-axis represents the methodological quality score (according to NOS), and the bubble size reflects the size of the sample size. Figure 3a showed the risk of caffeine intake (more than 100 mg/d) for infertility from 3 studies, all of them found no difference of incidence rate of infertility between low dose and no caffeine intake (OR varied from 0.77 to 1). Figure 3b showed the risk of caffeine intake (more than 200 mg/d) for infertility also from 3 studies. Both case-control studies found that the incidence of infertility was higher in the medium dose than no caffeine intake, but the cohort study did not show a different incidence of infertility between the two groups (OR varied from 0.67 to 1.64). Figure 3c showed the risk of caffeine intake (more than 400 mg/d) for infertility from 2 studies. The case-control study (OR 4.99) found that the incidence of infertility was higher in the high dose than no caffeine intake, but the cohort study did not show a different incidence of infertility between the two groups (OR 0.74).

**Fig. 4:**
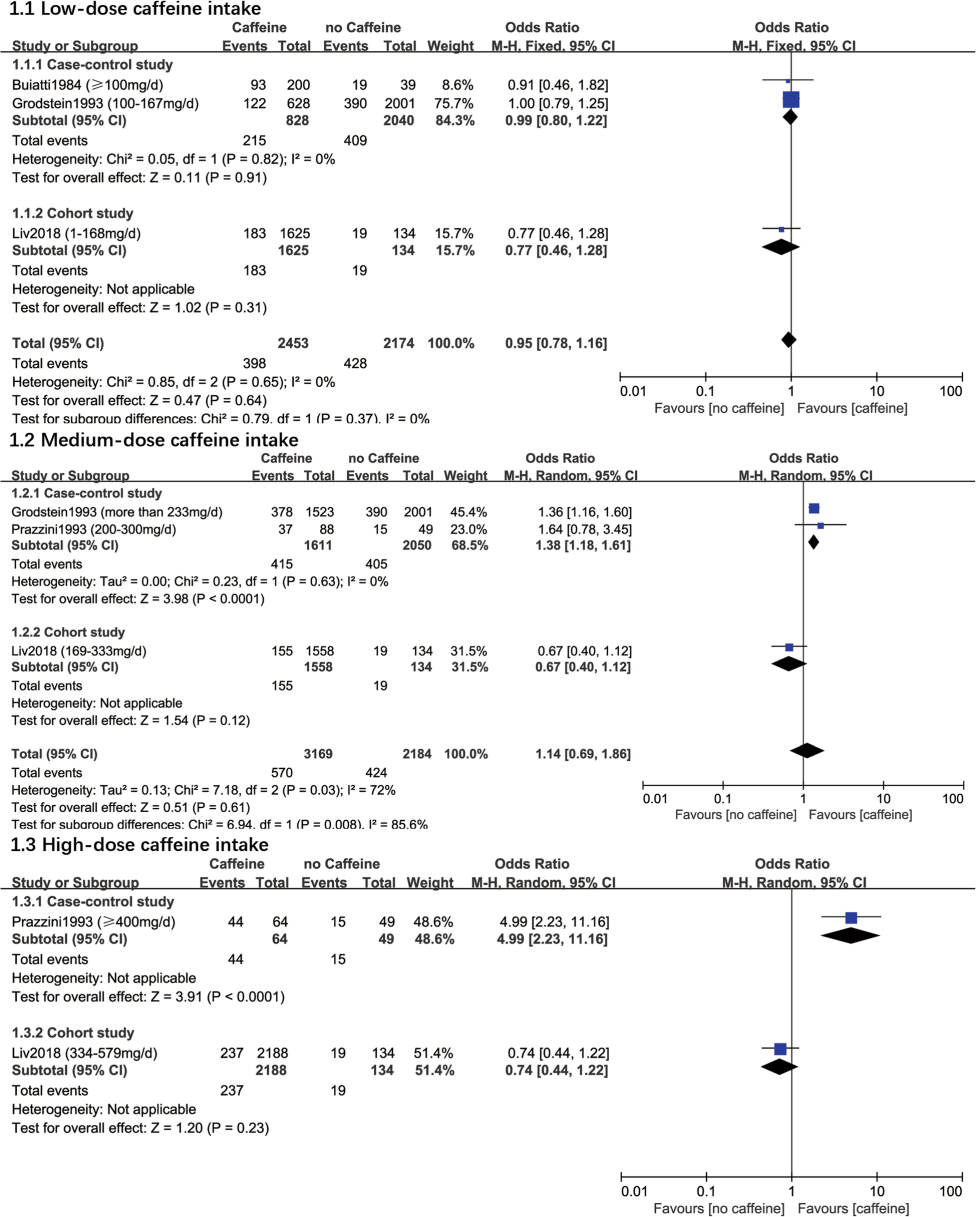
Forest plot of different dose caffeine intake and risk of infertility

Meta-analysis was also conducted (Fig. 4). Subgroup was classified according to the type of the study (case-control and cohort study). The results also showed caffeine intake may not increase the risk of infertility (lose dose: OR 0.95, 95%CI 0.78–1.16, M-H Fixed, *I*^*2*^ = 0%, *P* = 0.64, 4627 participants, 3 studies; medium dose: OR 1.14, 95%CI 0.69–1.86, M-H Random, *I*^*2*^ = 72%, *P* = 0.0006, 5353 participants, 3 studies; high dose: OR 1.86, 95%CI 0.28–12.22, M-H Random,*I*^*2*^ = 94%, *P* < 0.0001, 2435 participants, 2 studies). However, due to the obvious statistical heterogeneity when pooling the data for high dose of caffeine intake assessment (*I*^*2*^ = 94%,), results of this meta-analysis was only used for GRADE evaluation.

#### Quality of evidence

We used the GRADE method to evaluate the certainty of the evidence. For the three different doses of formed evidence bodies, no matter from which aspects, including large effect, dose-response gradient and plausible confounding, cannot be upgraded. The overall quality of the current evidence is all low as shown in Table 2.

**Table 2:** GRADE Summary of Findings Table

Caffeine intake	Large effect	Plausible confounding	Dose-response gradient	Anticipated absolute effects^a^ (95% CI)		Relative effect (95%CI)	No of Participants (studies)	Certainty of the evidence (GRADE)
				Risk with non caffeine	Risk with caffeine			
Low dose (≤100 mg/d)	No	No	No	197 per 1000	189 per 1000 (161 to 221)	OR 0.95 (0.78–1.16)	4627 (2case-control studies + 1 cohort study)	⊕ ⊕ ○○ Low
Medium dose (≥200 mg/d)	No	No	No	194 per 1000	215 per 1000 (143 to 309)	OR 1.14 (0.69–1.86)	5353 (2case-control studies + 1 cohort study)	⊕ ⊕ ○○ Low
High dose (≥400 mg/d)	No	No	No	186 per 1000	298 per 1000 (60 to 736)	OR 1.86 (0.28–12.22)	2435 (1case-control study + 1 cohort study)	⊕ ⊕ ○○ Low

^a^The risk in the intervention group (and its 95% confidence interval) is based on the assumed risk in the comparison group and the relative effect of the intervention (and its 95% CI)

*CI* Confidence interval, *OR* Odds ratio

GRADE Working Group grades of evidence

High certainty: We are very confident that the true effect lies close to that of the estimate of the effect

Moderate certainty: We are moderately confident in the effect estimate: The true effect is likely to be close to the estimate of the effect, but there is a possibility that it is substantially different

Low certainty: Our confidence in the effect estimate is limited: The true effect may be substantially different from the estimate of the effect

Very low certainty: We have very little confidence in the effect estimate: The true effect is likely to be substantially different from the estimate of effect

## Discussion

### Summary of evidence

Four studies [25–28] were included in this review. According to the NOS, the average score of the three case-control studies was 6, and the cohort study was 9. The sources of caffeine in the studies included coffee, tea, caffeine beverage (such as cola), cocoa and other drinks. Related confounding factors (such as age, smoking, drinking, history of obstetrics, etc.) were adjusted in three studies [26–28]. Low quality of the evidence showed caffeine intake may not increase the risk of infertility. Due to insufficient number of studies and insignificant statistical heterogeneity, we did not perform sensitivity analysis and subgroup analysis of participants’ characteristics.

Our study shows that the results of case-control studies and cohort studies are contradictory at high dose of caffeine intake. We note that the only cohort study included participants in the general population, while the three case-control studies included cases of infertility caused by various causes, such as azoospermia, oligospermia [25], dyspermia [27], ovulatory factor, tubal disease, cervical factor or endometriosis [26]. This may be due to the interaction effect of high-risk infertility factors (internal causes) and caffeine intake (external causes), which increase the risk of infertility.

### Compared with previous studies

Our previous meta-analysis [29] have found that caffeine intake during pregnancy may be associated with abortion, and the degree of risk increases with dose of the caffeine intake (> 300 mg/d, OR 1.35, 95%CI 1.27–1.44). The other published review [9] showed that caffeine intake might have a negative effect on male reproductive function by damaging sperm DNA. Compared to that systematic review our study concerned both male and female, and showed that caffeine intake did not appear increase the risk of infertility, regardless the doses of caffeine.

### Limitations and suggestions

Due to the limitations of the primary studies, we only get low-quality evidence to draw the conclusion. The four studies are all retrospective, and the analysis of the results requires consideration of the effects of recall bias, which may exist in the determination of exposure factors and the reporting of caffeine intake. More importantly, we have not found a recognized and objective method of measuring caffeine intake that for evidence synthesis. Therefore, we just referred to the method reported in the literature [4] as an operable measurement method. Although the results of most of the primary studies were obtained after adjusting for confounders, the comparability between the results also needed to be carefully considered due to the different approaches to dealing with confounders.

The quality of the evidence in our study is low, and one systematic review [30] indicates that low quality and retrospective studies are more likely to report the negative effects of caffeine on reproductive health. Therefore, high quality cohort and case-control studies are still needed to be designed in the future to explore the relationship between caffeine intake and infertility and theirs dose-response. Whether a cohort study or a case-control study, all important confounding factors should be considered as much as possible in the design stage, and they should be strictly controlled during implementation. In the analysis stage, statistical methods such as stratification and regression should be used to control and adjust confounding factors. For cohort studies, it is important to design a sufficiently long follow-up according to literature reports and clinical experience. For case-control studies, blinding surveys or interviews should be used whenever possible.

## Conclusions

Our study provides low quality evidence that regardless of low, medium and high doses of caffeine intake do not appear increase the risk of infertility. But we should treat this conclusion with caution.

## Supplementary information

**Additional file 1.** Search strategy in Pubmed. doc.

## Data Availability

All data generated or analyzed during this study are included in this published article and its supplementary information files.

## Abbreviations

CCB: Caffeine containing beverage
CCT: Controlled clinical studies
CI: Confidence interval
GRADE: The Grades of Recommendations Assessment, Development and Evaluation
HR: Hazard ratio
IVF: In vitro fertilization
MeSH: Medical Subject Headings
NOS: Newcastle-Ottawa Scale
OR: Odds ratio
RCTs: Randomized controlled trials
ROB: Cochrane risk of bias tool
SGA: Small for gestational age
