# Supplementary material for: Relationship between caffeine intake and infertility: a systematic review of controlled clinical studies
Source: BMC Womens Health. 2020 Jun 16;20:125. doi: 10.1186/s12905-020-00973-z (PMC7298863; doi:10.1186/s12905-020-00973-z)
Supplement: Supplementary file 1 — Additional file 1. Search strategy in Pubmed. doc. [file 12905_2020_973_MOESM1_ESM.docx]

**Appendix A Search Strategy in Pubmed.**

MEDLINE (via Pubmed)

#1 infertility[MeSH Terms]

#2 infertility[Title/Abstract]

#3 sterility[Title/Abstract]

#4 caffeine[MeSH Terms]

#5 caffeine[Title/Abstract]

#6 coffee[MeSH Terms]

#7 coffee[Title/Abstract]

#8 caffeinated[Title/Abstract]

#9 coffein[Title/Abstract]

#10 caffein[Title/Abstract]

#11 calcium caffeine[Title/Abstract]

#12 caffeine calcium complex[Title/Abstract]

#13 anhydrous caffeine[Title/Abstract]

#14 cafeine[Title/Abstract]

#15 animine[Title/Abstract]

#16 coffea[Title/Abstract]

#17 OR/#1-#3

#18 OR/#4-#16

#19 #17 AND #18
